# Engineering and Structural Insights of a Novel BBI-like Protease Inhibitor Livisin from the Frog Skin Secretion

**DOI:** 10.3390/toxins14040273

**Published:** 2022-04-12

**Authors:** Jie Yang, Chengliang Tong, Junmei Qi, Xiaoying Liao, Xiaokun Li, Xu Zhang, Mei Zhou, Lei Wang, Chengbang Ma, Xinping Xi, Tianbao Chen, Yitian Gao, Di Wu

**Affiliations:** 1 Chemical Biology Research Center, School of Pharmaceutical Sciences, Wenzhou Medical University, Wenzhou 325015, China; bkaqgo@163.com (J.Y.); tcl1173148508@163.com (C.T.); 184511382270@stu.wzu.edu.cn (X.L.); 2 College of Life and Environmental Science, Wenzhou University, Wenzhou 325035, China; qjm1357@163.com (J.Q.); 194611372150@stu.wzu.edu.cn (X.L.); zhangx24@wzu.edu.cn (X.Z.); 3 Natural Drug Discovery Group, School of Pharmacy, Queen’s University Belfast, Belfast BT7 1NN, UK; m.zhou@qub.ac.uk (M.Z.); l.wang@qub.ac.uk (L.W.); c.ma@qub.ac.uk (C.M.); x.xi@qub.ac.uk (X.X.); t.chen@qub.ac.uk (T.C.)

**Keywords:** protease inhibitor, antimicrobial peptide, natural product, skin secretions, animal venoms

## Abstract

The Bowman–Birk protease inhibitor (BBI) family is a prototype group found mainly in plants, particularly grasses and legumes, which have been subjected to decades of study. Recently, the discovery of attenuated peptides containing the canonical Bowman–Birk protease inhibitory motif has been detected in the skin secretions of amphibians, mainly from Ranidae family members. The roles of these peptides in amphibian defense have been proposed to work cooperatively with antimicrobial peptides and reduce peptide degradation. A novel trypsin inhibitory peptide, named livisin, was found in the skin secretion of the green cascade frog, *Odorrana livida*. The cDNA encoding the precursor of livisin was cloned, and the predicted mature peptide was characterized. The mature peptide was found to act as a potent inhibitor against several serine proteases. A comparative activity study among the native peptide and its engineered analogs was performed, and the influence of the P_1_ and P_2′_ positions, as well as the C-terminal amidation on the structure–activity relationship for livisin, was illustrated. The findings demonstrated that livisin might serve as a potential drug discovery/development tool.

## 1. Introduction

Amphibians possess chemically complex skin secretions to protect them from predators and pathogens. The peptides present in these skin secretions are primarily responsible for the antimicrobial activities of the skin secretions. The naturally occurring antimicrobial peptides (AMPs) are repositories for new antibiotic discovery and development. The most common mechanism of these AMPs is to disrupt and permeabilize the cell membrane of microorganisms. As the microbial membrane composition is evolutionarily stable, microorganisms are unlikely to acquire resistance to such AMPs through mutation [1]. Due to the unique antimicrobial mechanism, AMPs have become an attractive source of drug research. To date, more than one third of naturally occurring active peptides are from amphibians [2]. However, there are still some obstacles to applying them clinically. A key reason is that most of the peptides contain charged groups contributed by positively charged amino acid residues such as lysine and arginine, which make the AMPs susceptible to degradation by trypsin or trypsin-like proteases. Identifying peptides as protease inhibitors in amphibian skin secretion would have great significance as these peptides play an important role in the preservation of the host through evolution. Their role in amphibian defense has been proposed to work cooperatively with other antimicrobial peptides to reduce peptide degradation [3].

To date, many peptidic serine protease inhibitors have been identified in amphibian skin and skin secretion. The Kunitz-type serine protease inhibitors were discovered in the Sambava tomato frog (*Dyscophus guineti*) [4]. The Kazal-type inhibitors were found in the Red-eyed tree frog (*Agalychnis callidryas*) [5]. Several Bowman–Birk-like inhibitors, such as HV-BBI [3], HJTI [6], and pLR-HL [7], were also found in frogs. Serine proteases catalyze various physiological processes, such as food digestion, blood coagulation, immune defense, cell death, tissue remodeling, proenzyme activation, and so on [8]. When imbalances of homeostasis between proteases and their endogenous inhibitors occur, uncontrolled proteolysis may be triggered in the internal environment, leading to pathophysiological reactions such as gastric ulcers, hypertension, inflammation processes, and tumor growth [9]. Serine proteases are involved in various diseases’ pathogenesis, and their inhibitors can be ideal drug leads as inhibitors for these proteases.

In this study, a novel BBI-like trypsin inhibitor precursor was cloned using 3′-RACE from the skin secretion of the green cascade frog (*Odorrana livida*). The mature peptide was isolated, characterized, synthesized, and named livisin (*O. livida* serine protease inhibitor). Moreover, the native peptide was modified, mainly by substituting the P_1_ residue, to obtain four livisin analogs. The synthetic peptides were subjected to functional assays to evaluate their bioactivities and initial structure–activity relationships (SAR).

## 2. Results

### 2.1. Cloning of the Livisin Precursor-Encoding cDNA and Structural Characterization of Livisin

**Figure 1:**
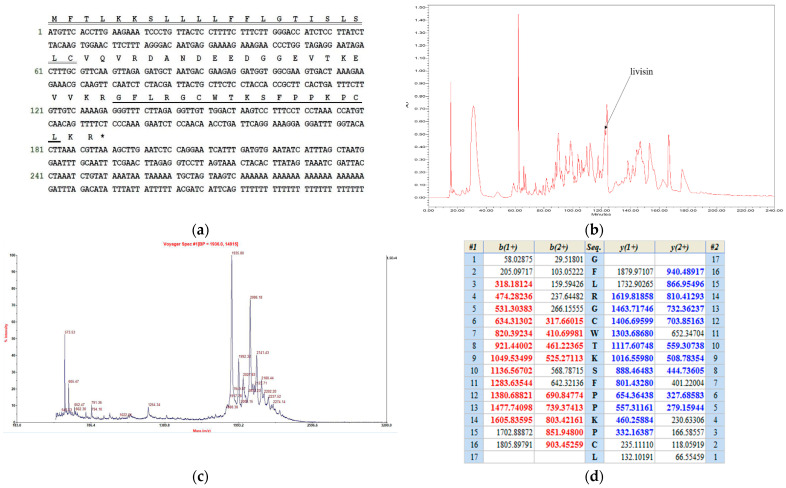
Identification and characterization of livisin. (**a**)The nucleotide sequence (Genbank: LT591895) of the cDNA cloned from *O. livida* skin secretion and its predicted peptide sequence. The signal peptide (double-underlined), putative final mature peptide (single-underlined), and stop codon (asterisk) are indicated. (**b**) Relevant region of RP-HPLC chromatogram of *O. livida* skin secretion at 214 nm with an arrow indicating the elution position/retention time of livisin. (**c**) MALDI-TOF mass spectrum of the RP-HPLC fraction containing the *m/z* 1935.8 compound. (**d**) MS/MS fragmentation data of fragment ions corresponding to those of livisin. Expected singly and doubly charged b-ion and y-ion fragment *m/z* ratios were predicted online through Protein Prospector (http://prospector.ucsf.edu/prospector, accessed on 16 April 2016). Observed fragment ions are indicated in a colored bold typeface.

A pair of primers DV-3, which was designed to be complementary to the highly conserved 5′-UTR of AMP precursor cDNAs that were previously identified from other Rana frogs, and NUP (nested universal primer) was employed to perform the 3′-RACE. Through 3′-RACE, the livisin precursor encoding cDNA was cloned from the *O. livida* skin secretion-derived cDNA library. The cDNA was found to contain an open reading frame coding for 63 amino acid residues, presumably the precursor (Figure 1a). It comprised a signal peptide of 22 residues terminating with cysteine, a propeptide of 20 residues acting as an acidic spacer, and a lysine–arginine (KR) cleavage site followed by a putative 19-mer mature peptide and a C-terminal KR cleavage site. Therefore, we deduced that the final mature peptide might end with a leucine residue. Alignment of the mature livisin sequence with other Bowman–Birk-like inhibitors revealed the conserved reactive disulfide loop typical of the Bowman–Birk-like inhibitors [10].

Furthermore, the skin secretion was fractionated by reverse-phase HPLC (RP-HPLC) (Figure 1b) followed by identification of the different peaks by mass spectrometry. A peptide with *m/z* 1935.8, consistently identical to the putative mature livisin, was found in the RP-HPLC fraction (Figure 1c), thereby confirming the prediction that the final mature peptide is a cleaved product without the C-terminal KR. The primary structure of the mature peptide was further characterized by MS/MS fragmentation sequencing (Figure 1d). This novel peptide was identified as having a canonical Bowman–Birk inhibitor (BBI) geometry, which contained an antiparallel disulfide bridge.

### 2.2. Design, Synthesis, and Secondary Structure Confirmation of Livisin and Analogs

#### 2.2.1. Design and Synthesis of the Native Livisin and Analogs

The trypsin catalytic triad serine interacts with serine protease substrate, resulting in the hydrolysis of the P_1_-P_1′_ peptide bond. Generally, the P_1_ residue in the inhibitory binding loop plays a crucial role in the inhibitory potency and selectivity of the protease. We substituted the P_1_ residue lysine in livisin with arginine or phenylalanine to yield livisin-Arg and livisin-Phe, respectively. The C-terminal amidation of AMPs could effectively improve the resistance of the peptides against carboxyl peptidase cleavage and, hence, enhance its antimicrobial activity. Thus, we modified livisin by C-terminal amidation to livisin-NH_2_. Furthermore, a truncated form of livisin that contained only the inhibitory loop was also obtained to investigate the contribution of the flanking residues. Each of the peptides contained two cysteine residues that formed a disulfide bond. Livisin and its analogs were synthesized by a solid-phase peptide synthesizer using the Fmoc method, oxidized by dimethyl sulfoxide (DMSO), and purified by RP-HPLC. The purity and authenticity of the structures were confirmed by MALDI-TOF and analyzed by MS/MS fragmentation. 

#### 2.2.2. Confirmation of the Secondary Structures of Native Livisin and Analogs by Circular Dichroism

**Figure 2:**
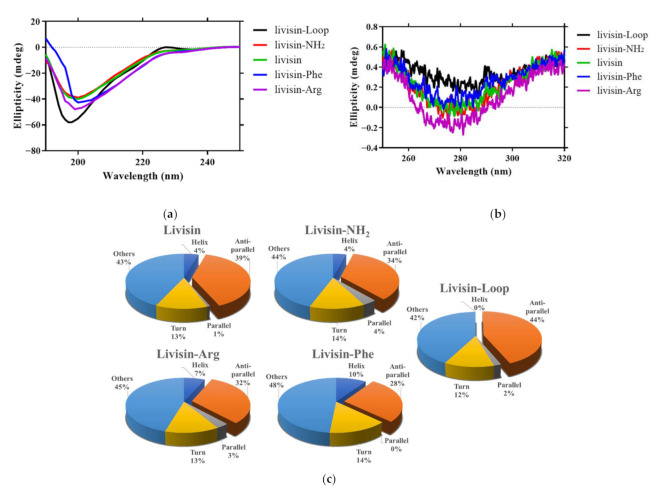
CD spectra of native livisin and analogs. (**a**) Far-UV CD spectra of native livisin and analogs. (**b**) Near-UV spectra of native livisin and analogs. (**c**) The secondary structure compositions of native livisin and analogs were predicted and calculated by the BeStSel webserver.

Circular dichroism (CD) spectroscopy is widely used to analyze peptide conformation. The far-UV spectra of livisin and its various modified forms displayed negative bands around 200 nm (Figure 2a), reminiscent of no dominant secondary structures [11]. The near-UV region of the spectra was attributed to the aromatic residues and the disulfide bonds. The peptides showed negative peaks around 280 nm in the near-UV spectra (Figure 2b). Compared with native livisin, livisin-NH_2_ displayed no significant conformational difference in amplitude, while livisin-Arg and livisin-loop displayed strong spectral amplitudes and a blue shift in the far-UV spectrum. In contrast, a different trend of the spectrum of livisin-Phe was observed, indicating the potential connection between conformation change and specificity for switching protease inhibitory activities. By using the CD spectra data, the secondary structure compositions of native livisin and analogs were calculated using the BeStSel web server [12]. The percentages of antiparallel β-sheet in livisin, livisin-NH_2,_ livisin-Arg, livisin-loop, and livisin-Phe were 39%, 34%, 32%, 44%, and 28%, respectively (Figure 2c).

### 2.3. Effects of Synthetic Livisin and Its Analogs on Protease Inhibition

**Figure 3:**
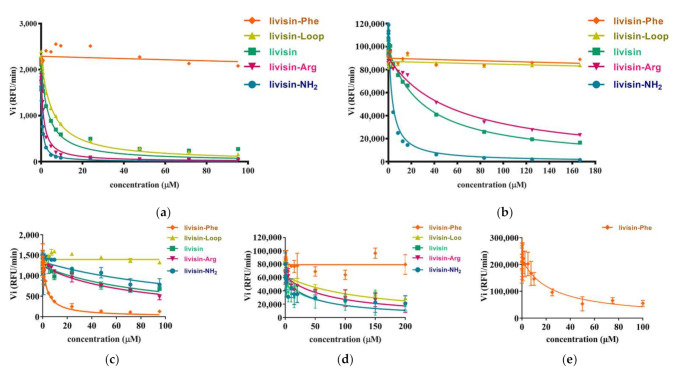
Inhibitory effects of livisin and its modified forms on the activities of various serine proteases. (**a**) Bovine trypsin; (**b**) matriptase; (**c**) chymotrypsin; (**d**) mesotrypsin; (**e**) human proteasome 20s. The activity of each protease was assayed in the presence of each peptide ranging from 0.1 μM to 100 μM in PBS (pH 7.4) and the corresponding substrate: Z-Gly-Gly-Arg-NHMec (trypsin); Suc-Ala-Ala-Pro-Phe-NHMec (chymotrypsin); Boc-Gln-Ala-Arg-AMC (matriptase); Mca-Arg-Pro-Val-Glu-Nval-Trp-Arg-Lys(Dnp)-NH_2_ (mesotrypsin); Suc-Leu-Leu-Val-Tyr-AMC (human proteasome 20s).

As most canonical BBI peptides can inhibit serine protease activities, the inhibitory activities against different proteases (bovine trypsin, chymotrypsin, matriptase, mesotrypsin, and human proteasome 20s) of the native livisin and analogs were assessed. The inhibitory activities against these proteases were tested at a peptide concentration ranging from 0.1 μM to 100 μM. The Ki (inhibition constant) value of each peptide was calculated and is summarized in Table 1, and the Morrison plots of peptides acting on the proteases are shown in Figure 3. The native livisin possessed moderate trypsin inhibitory activity (Ki = 2.792 μM), but amidation of the peptide at the C-terminus increased the inhibitory activity 7-fold (Ki = 0.424 μM). Livisin-Arg, in which the P_1_ residue was substituted with arginine and showed similar inhibitory activity (Ki = 1.016 μM), but the peptide livisin-loop showed lower potency (Ki = 4.252 μM). Neither livisin nor the analogs (livisin-NH_2_, livisin-Arg, and livisin-loop) exhibited good inhibitory activity against chymotrypsin. The peptide livisin-Phe, in which the P_1_ residue was replaced by phenylalanine, turned out to effect a moderate chymotrypsin inhibition (Ki = 2.362 μM) instead of trypsin inhibition. Consistent with chymotrypsin-inhibitory activity, only livisin-Phe showed inhibitory effects against the human 20s proteasome, with a Ki value of 17.72 μM. The livisin and its analogs were further tested for the inhibition of human matriptase-1 with trypsin-like substrate specificity. The activities of these peptides against these two proteases were comparable to that against trypsin, with only minor differences. The amide-modified livisin-NH_2_ was the most effective against human matriptase. Its Ki value (2.362 μM) was approximately one sixth of trypsin inhibition (0.4242 μM). The natural livisin and livisin-Arg maintained moderate inhibitory activities, approximately ten and twenty times higher than livisin-NH_2_, respectively. Livisin-Phe and livisin-loop completely lost their matriptase-inhibitory activity. Although mesotrypsin shares high sequence identity with trypsin, the proteinaceous trypsin inhibitors are less effective. It was reported that the residue of the P_2′_ position of plant-derived cyclic peptides was important for the specificity of mesotrypsin, and aromatic residues were the most favored for mesotrypsin inhibition [13]. As livisin and its derived peptides possess the phenylalanine at the P_2′_ position, we further explored their inhibitory activity against mesotrypsin. Unexpectedly, natural livisin and its analogs showed weaker inhibition against mesotrypsin compared with their effects against bovine trypsin.

### 2.4. Antimicrobial/Hemolytic Activities of the Peptides

The antimicrobial activities of the five peptides were assessed by determining their minimal inhibitory concentrations (MICs) against three standard microorganisms, the Gram-positive bacterium *Staphylococcus aureus* (NCTC 10788) and the Gram-negative bacterium *Escherichia coli* (NCTC 10418), and the opportunistic pathogen yeast *Candida albicans* (NCPF 1467). Except for livisin-NH_2_, which showed weak inhibition against *C. albicans* with an MIC of 256 μg/mL (132 μM), all the other peptides were devoid of antimicrobial activities at the highest concentration tested (Table 2).

**Figure 4:**
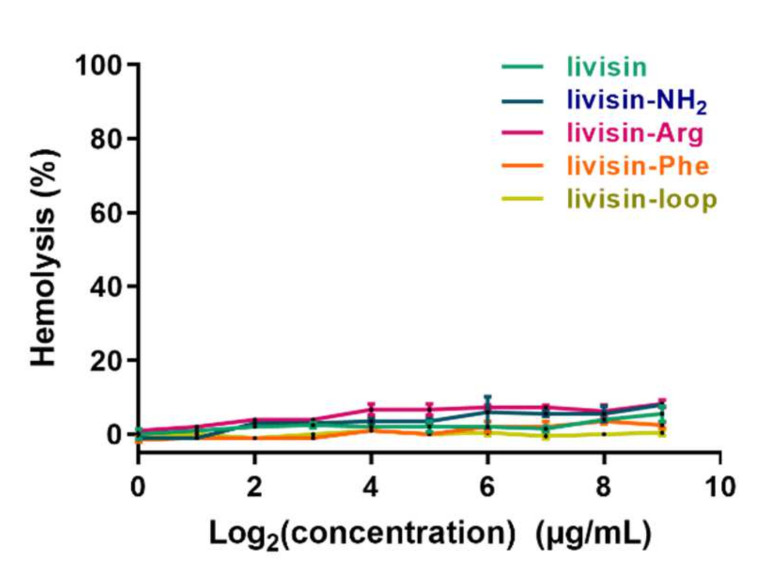
Hemolytic activity of the native livisin and analogs. The tested concentrations of the peptides range from 1 μg/mL to 512 μg/mL.

The hemolysis percentage of each peptide was calculated. All peptides showed less than 8% hemolysis at the highest tested concentration (512 μg/mL) (Figure 4). The weak hemolytic toxicity mirrored their weak antimicrobial activity.

### 2.5. Molecular Docking and Structure–Activity Relationships of Livisin and the Analogs

**Figure 5:**
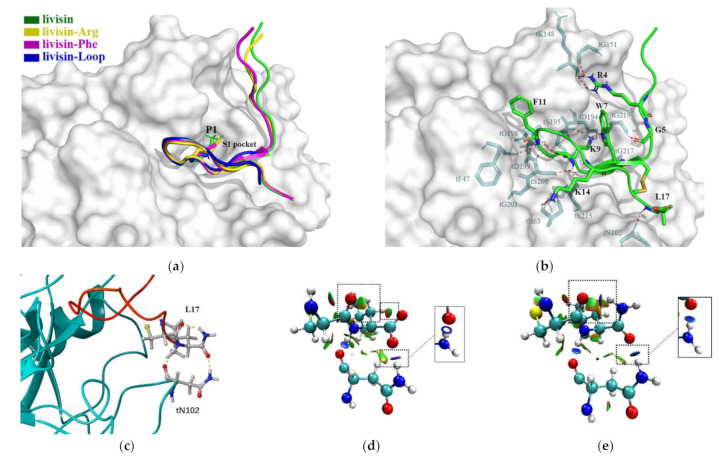
Molecular docking of livisin and its analogs and bovine trypsin. (**a**) Comparative docking result of livisin analogs and trypsin. Livisin, livisin-Arg, livisin-Phe, and livisin-loop are shown in green, yellow, pink, and blue color and are represented by cartoons. P_1_ residues of these peptides are represented by sticks and are inserted into the trypsin S1 pocket. (**b**) Detailed polar interactions between livisin and trypsin. Trypsin is shown as the surface and livisin is represented by the cartoon in green color with the disulfide bond in yellow. The residues in livisin and trypsin that have polar interactions are represented by sticks with green and cyan colors, respectively, and the interactions are rendered in red dots. The oxygen, nitrogen, and hydrogen atoms are shown as red, blue, and white in livisin. (**c**) Interactions between the L17 of livisin-NH_2_ and tN102 of trypsin (stick representation); livisin-NH_2_ is shown in a red cartoon. (**d**,**e**) Three-dimensional maps of intermolecular and intramolecular interactions around C16 and L17 of livisin (**d**), livisin-NH_2_ (**e**) (**top**), and tN102 (**bottom**) of trypsin, as demonstrated by the noncovalent interaction (NCI) analysis [14]; the main differences are framed.

We further investigated the enzyme-inhibitor binding mode. The peptide models were simulated by the I-TASSER server and were validated by ProSA. Generally, livisin and the analogs adopted a canonical pose of the BBI family in the enzyme-inhibitor complex with the side chain of the P_1_ residue deeply inserted into the S1 pocket of bovine trypsin (Figure 5a). Using the native peptide livisin as an example (Figure 5b), the hydrogen bonds are formed by the side chain amine of livisin K9 and the carbonyl of main chain trypsin S195 (tS195), tG219, as well as the electronic attraction with the side chain of tD194. Livisin K9 contributes the most significant number of hydrogen bonds in the complex. Moreover, the hydrogen bonds are formed between the main chain carbonyl oxygen of livisin K9 and the main chains of three successive residues of trypsin (tG198, tD199, and tS200). Moreover, the main chain amide of K9 also donates hydrogen bonds to the side chain hydroxy group of tS200 and the backbone carbonyl oxygen of tS215. In general, livisin could adopt a substrate-like manner and interact with the catalytic site of trypsin but without forming any covalent bond. This suggests that livisin behaves as a competitive reversible inhibitor of trypsin.

The side chain of the other basic residue K14 donates a hydrogen bond with the tH63 main chain. Within the inhibitory loop, the main chain amide of F11 donates a hydrogen bond to the main chain carbonyl oxygen of tF47. W7 of livisin and tG217 resembles an antiparallel β-sheet conformation stabilized by the two hydrogen bonds between their backbones. Moreover, the W7 side chain of livisin forms a hydrogen bond with another glycine, which is tG219.

The residues outside of the inhibitory loop also contribute to the complex interaction. The N-terminal R4 residue of livisin donates two hydrogen bonds with its side chain guanidine group to the backbone carbonyl of tK148 and tG151. The livisin G5 adjacent to the inhibitory loop forms a hydrogen bond with tG219. For the C-terminus, the backbone amide of the last hydrophobic residue, L17, participates in a hydrogen bond with the backbone carbonyl of tN102.

We further assessed the interaction differences between the native livisin and its amide-modified peptide livisin-NH_2_. The electron densities of both peptides were almost the same, but an extra intracellular hydrogen bond was formed between the oxygen atom of C16 and the hydrogen atom of L17 in livisin-NH_2_. Additionally, another hydrogen bond was formed between the oxygen of the final amide of livisin-NH_2_ and the side chain of tN102 of trypsin (Figure 5c).

For further confirmation, the noncovalent interactions between livisin and trypsin were analyzed. Compared to the native livisin (Figure 5d), the amide modification (Figure 5e) did change the interactions. Except for the formation of the polar interaction of the aforementioned hydrogen bonds, the vanished repulsion and Van der Waals’ force interaction were also observed.

## 3. Discussion

**Figure 6:**
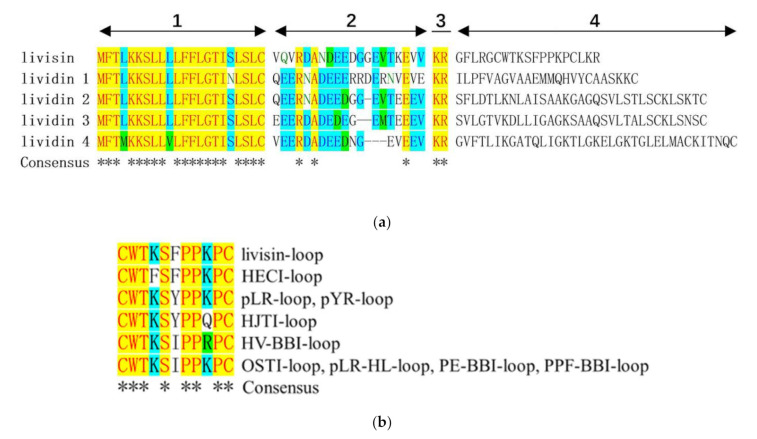
Alignment of livisin amino acid sequences. (**a**) Alignment of livisin precursor and lividin precursors (the variable mature peptides are not aligned). The domains of the precursors are indicated: (1) signal peptide region, (2) acidic spacer peptide region, (3) KR endoproteolytic processing site, (4) hyper mutable functional peptide region. (**b**) Alignment of the conventional inhibitory loop of the identified natural BBI-like peptides. Consensus residues are labeled in red letters with yellow background and indicated by asterisks. Conservative residues are highlighted by the light blue background, and similar residues are indicated by green background. Alignment was prepared using the ClustalW algorithm in MEGA11 [25] and colored.

As a typical potent serine protease inhibitor family, the Bowman–Birk inhibitors have been identified in many plants, especially in leguminous and gramineous plants [15,16], and these peptide inhibitors typically consist of nine amino acid residues having the sequence CTP_1_SXPPXC, where P_1_ is the residue that is subject to cleavage [10]. Intriguingly, the amphibian Bowman–Birk-like inhibitors are attenuated peptides containing the canonical Bowman–Birk protease inhibitory motif, which consists of eleven residues, CWTP_1_SXPPXPC. The binding loop of amphibian BBI is two amino acid residues longer than the plant BBI loop (e.g., sunflower trypsin inhibitor, SFTI). Since the plant BBIs are located closer to the active site of the protease, they may elicit more potent activity than the amphibian BBI [10]. Additionally, the plant BBIs are present in high abundance in the seeds, and they probably act as storage proteins. It has been proposed that the ancestral proteins in plants were short of a protease inhibitory function, and the protein-folding scaffolds were recruited as protease inhibitors. A similar situation probably applied to frog skin BBIs, which may have evolved from a typical AMP. Lividins were previously identified as AMPs from *O. livida*, and they belong to the potent antimicrobial brevinin, nigrocin, and esculentin families [17]. The homology between livisin and lividin can be illustrated by the high conservation in both the signal peptide and the acidic spacer region between the two precursors (Figure 6a). The hypermutation of the mature peptide domain may be explained by the evolution of a selective survival value to adapt to different environments and resist predators or microorganisms [18]. The evolutionary pressure for amphibians to produce protease inhibitors in response to invasive exogenous proteases secreted by predators or pathogens could therefore be part of an “evolutionary arms race”. Moreover, the inhibitors might also modulate the processing of the secreted peptide precursors and prevent the peptides from being degraded [19].

Although BBIs are highly conservative, the structure of livisin still contains unique features, especially the P_2′_. Some studies considered that the P_2′_ residue of the SFTI was associated with potency and selectivity [20]. As shown in Figure 6b, the P_2′_ residue of the conventional inhibitory loop of the identified natural BBI-like peptides could be isoleucine, tyrosine, or phenylalanine [3,6,7,21,22,23]. The frequency of Ile at P_2′_ is high, but only Phe was found at P_2′_ in the livisin-loop and HECI-loop. Some research indicates that the P_2′_ residue may relate to the specificity of the BBI peptides. The crystal structure of a previously discovered HV-BBI peptide from Chinese bamboo odorous frog *Huia versabilis* and bovine trypsin has been determined [24]. Within the HV-BBI (3-18) loop, the only other insertion involved in the complex is IleP_2′_. The side chain of IleP_2′_ is inserted into a shallow pocket of the trypsin. The molecular docking of livisin to trypsin demonstrates a similar insertion. PheP_2′_ inserts into the shallow curve with a hydrogen bond to the tF47 of trypsin. Compared with the short side-chained isoleucine at the P_2′_ position in HV-BBI, both the phenylalanine residue in livisin and a tyrosine residue in HJTI are aromatic, which may abolish the fragile affinity because of steric hindrance. We need further experiments to show how the P_2′_ residue could affect the activity of the peptide.

The presence of phenylalanine at residue 2 and 11 of the livisin and its analogs (livisin-Arg, livisin-Phe, livisin-NH_2_) may also affect their CD spectra. It is reported that a two-stranded antiparallel β-sheet composed the active inhibitory loop in SFTI [26]. However, the livisin analogs showed no dominant secondary structures on the CD spectra, probably because of the relatively higher content of aromatic residues. Woody et al. found that, in the case of BPTI (bovine pancreatic trypsin inhibitor), which has four phenylalanine and four tyrosine residues, the CD spectrum calculated from only the peptide transitions differed a great deal from the experimental CD. The experimental CD spectra could not display typical peaks of β-sheet structures, while the calculated CD results were close to the real conformation [27]. Interestingly, the experimental CD of BPTI has a negative peak at 200 nm, a shoulder at 220 nm, and a significant negative peak at 280 nm [27], which are also the characteristic peaks of the livisin analogs. Although the far-UV spectra did not show a typical β-sheet character, livisin and its derived peptides were predicted to contain around 30% antiparallel β-sheet. Livisin-loop has a higher content of β-sheet (44%) than livisin and other analogs, and this may be because of the lack of the flanking residues that could adopt an α-helix or unpacked conformation. In addition, livisin-Phe has the lowest content of β-sheet and this may be caused by the additional aromatic residue in the inhibitory loop, which could affect its CD spectrum.

The inhibitory activities of livisin against several serine proteases were explored to study the structure–activity relationships. Natural livisin displayed the most potent inhibition against trypsin (2.792 μM) but exerted only moderate inhibition against mesotrypsin (22.38 μM), matriptase (25.15 μM), and chymotrypsin (92.71 μM), while showing no detectable inhibition against the human 20s proteasome. Following assessment of the inhibitory activities of livisin, different strategies were employed to design livisin analogs for the initial SAR study. First, livisin was C-terminally amidated to investigate the effect of C-terminal amidation. Next, K9 of livisin was substituted by arginine and phenylalanine to investigate the function of the reactive P_1_ residue. Finally, a truncated form of the livisin was made that contained only the reactive loop for a comparative activity study. As shown in the result, the K9 inserts into the pronounced trypsin S1 pocket and the inhibitor acts in a substrate-like manner. It contributes the most significant number of hydrogen bonds in the complex. Some research indicated that the guanidium moiety of arginine is likely to be more favorable in interactions with the solvent [24]. The result that the analog livisin-Arg displayed similar inhibitory activity may be because the side chain of arginine residue is long enough to insert into the S1 pocket and form hydrogen bonds (Figure 5a). However, the guanidine group at the end of the side chain of arginine is larger than the amine group from the side chain of lysine. Therefore, we deduced that the stereo hindrance of arginine in livisin-Arg might be responsible for the two-fold decrease in trypsin-inhibitory activity. Combining the docking results, the backbone of the phenylalanine in livisin-Phe could still form the hydrogen bonds to trypsin, similarly to livisin, but the side chain of phenylalanine is not involved in any hydrogen bond contacts. Therefore, the substitution of K9 with phenylalanine might have eliminated the trypsin-inhibitory activity (Ki > 300 μM). However, the converted chymotrypsin-inhibitory activity exhibited (Ki = 2.706 μM) was in line with our expectation. The substrate-like model accounts for transforming the inhibitory specificity as chymotrypsin preferentially acts on aromatic amino acid residues such as phenylalanine, tyrosine, and tryptophan. The 20s proteasome is a highly conserved proteasome to maintain cellular homeostasis, including neuronal communication, post-translational processing, oxidative stress, intrinsically disordered protein regulation, and extracellular proteasomes. The conserved architecture is composed of 28 subunits with caspase-like activity (β1), trypsin-like activity (β2), and chymotrypsin-like (β5) functions. Consistent with chymotrypsin-inhibitory activity, only livisin-Phe showed inhibitory effects against the human 20s proteasome, with the Ki value of 17.72 μM.

Compared to the natural livisin, livisin-NH_2_ could form an intramolecular hydrogen bond between the oxygen of C16 and hydrogen of L17. The conformation stretches caused by the intramolecular hydrogen bond in the livisin-NH_2_ led to another hydrogen bond formation between the terminal amide of L17 and the tN102 of trypsin. As observed in the analysis of the noncovalent interactions, the additional hydrogen bond of livisin-NH_2_, together with the vanished repulsion and altered Van der Waals’ force interaction, may account for the seven-times increase in trypsin-inhibitory activity. Except for the canonical loop as the active core, the flanks adjacent to the inhibitory loop are also important for maintaining the inhibitory properties. Similar to a safety belt, both residues G5 and L17 on the flanks adjacent to the inhibitory loop formed hydrogen bonds to trypsin and contributed to the affinity significantly. Except for G5 and L17, R4 also donates hydrogen bonds to trypsin.

The other residues P_6_ and P_9′_ outside of the loop point away and do not interact with the enzyme. These residues may be redundant for the inhibitory activity of the BBI peptides. Przemyslaw et al. also showed that the selective deletion of these residues is feasible for simplifying the inhibitors. The deletion of P_7_ and P_8_ of HV-BBI leads to HV-BBI (3-18) and their inhibitory activities being almost unchanged. However, the removal of P_8_ and P_11′_ together from ORB increased the activity by 408 times. Moreover, the continued removal of P_7_ increased the activity by 1 × 10^5^ times, compared with ORB [24,28].

Livisin and its analogs did not demonstrate potent antimicrobial activities under the tested concentrations. Only livisin-NH_2_ displayed a weak effect against *C. albicans*, with an MIC value of 256 μg/mL (132 µM). The C-terminally amidated peptide has one more positive charge than the native one with free C-termini, which may contribute to the affinity between the peptide and negatively charged microbial membrane, and it is well established that the amidated modification on the C-termini of AMPs could eliminate or slow proteolytic degradation to prolong the stability of peptides [29]. We know that the inhibitory loops are devoid of microbial killing [6,7,30]. These facts suggest that the inhibitory loop is essential for trypsin inhibition, but the residues outside the loop contribute to certain peptides’ antimicrobial activity.

Following the outbreak of the COVID-19 epidemic, we were willing to make efforts in anti-virus drug research and development. The bi-functional BBI-like peptides seem to be good leads because serine proteases are involved in many viruses’ life cycles. The molecular-level viral protease inhibitory activity screening will be carried out in our future work.

## 4. Materials and Methods

Acquisition of skin secretion sample. Specimens of the green cascade frog (*O. livida*) (*n* = 3, snout-to-vent length 8–10 cm, adults, sex undetermined) were captured in the field around the city of Fuzhou, Fujian Province, China. The secretion was harvested in the field and then the frogs were released. Skin secretion was obtained by mild transdermal electrical stimulation, as previously described [31], because it does not harm the frogs and exerts minimal pressure on them. The secretions were collected and subsequently frozen in liquid nitrogen and later lyophilized. The lyophilizate was stored at −20 °C.

“Shotgun” cloning of skin secretion peptide precursor-encoding cDNAs. Lyophilized frog skin secretion (5 mg) was dissolved in 1 mL of cell lysis/binding buffer (Dynal Biotech, UK) and vortexed un-continuously. Polyadenylated mRNA was trapped and isolated by oligo-dT magnetic beads and was reverse-transcribed to obtain skin secretion cDNA library. The nested universal primer (NUP: 5′-AAGCAGTGGTATCAACGCAGAGT-3′) was supplied with a SMART-RACE kit (Clontech, Palo Alto, CA, USA), and the degenerated DV-3 (5′-GAWYYAYYHRAGCCYAAADATG-3′) was employed as a pair of primers to perform the 3′-RACE to obtain peptide precursor nucleic acid sequence data. DV-3 was designed to be complementary to the highly conserved 5′-UTR of AMP precursor cDNAs that were previously identified from other Rana frogs. The 3′-RACE products were purified by use of the Cycle-Pure Kit (Omega Bio-Tek, Norcross, GA, USA), cloned through a pGEM-T vector system (Promega, Southampton, UK), and sequenced using an ABI 3100 sequencer. The bioinformatic investigation was carried out by submitting the peptide precursor sequence to the Basic Local Alignment Search Tool (BLAST) of the National Centre for Biotechnology Information (NCBI). The nucleotide sequence of the cDNA encoding the novel livisin precursor from the skin secretion of *O. livida* has been deposited in the EMBL Nucleotide Sequence Database under the accession code LT591895.

Identification and structural analysis of the mature peptide in skin secretion. Lyophilized skin secretion powder sample (10 mg) was dissolved in 1.5 mL TFA/water (0.05/99.5, *v*/*v*) and then clarified by centrifugation. The clarified supernatant (1 mL) was injected into an RP-HPLC system (Waters, Milford, MA, USA) with an RP column (Vydac, C4, 300 Å, 5 μm, 4.6 mm i.d. × 250 mm, Grace Vydac, Deerfield, IL, USA) using a gradient program from TFA/water (0.05/99.5, *v*/*v*) to TFA/water/acetonitrile (0.05/29.95/70.0, *v*/*v*/*v*) in 240 min at 1 mL/min flow rate under monitoring of bi-wavelength (λ = 214 nm and λ = 280 nm). The fractionated elutes were collected at 1 min intervals. The molecular masses of contents in each fraction were analyzed by an MALDI-TOF mass spectrometer (PerSeptive Biosystems, Forster City, CA, USA) in positive detection mode using α-cyano-4-hydroxycinnamic Acid (CHCA) as the matrix. The fraction with peptide molecular mass coincident with the predicted mature peptide from cloned cDNA was then injected into an LCQ Fleet^TM^ ion-trap electrospray mass spectrometer (Thermo Fisher Scientific, San Francisco, CA, USA) for MS/MS fragmentation sequencing.

Solid-phase peptide synthesis. Following the confirmation of the primary structure of the cloned cDNA-encoded peptide, livisin and its analogs were chemically synthesized by a Tribute^TM^ automated solid-phase peptide synthesizer (Protein Technologies, Tucson, AZ, USA). Then, the peptides were de-protected and cleaved from the resin. For the formation of the disulfide bond, an extra oxidation step was performed. Briefly, the washed crude peptide precipitate was suspended in diethyl ether, and several drops of DMSO were added and stirred at room temperature for three days. An Adept CE 4200 (Cecil Instrument, Cambridge, UK) RP-HPLC was used for the purification of chemically synthesized peptides. A gradient program was achieved by using two different mobile phases, which were prepared as follows: mobile phase A: trifluoroacetic acid (TFA)/water (0.05/99.95, *v*/*v*); mobile phase B: TFA/water/acetonitrile (0.05%/19.95/80.00, *v*/*v*/*v*). The target elute was collected and lyophilized to obtain the purified peptides. The purity and authenticity of structures were confirmed by HPLC and MS.

Kinetic assays for the inhibition constant (Ki). Livisin and variants were screened against trypsin, chymotrypsin, matriptase, mesotrypsin, and human 20s proteasome, respectively. Inhibitory activities against the proteases of the peptides were tested using previously published papers [22] and manufacturers’ instructions from 0.1 μM to 100 μM in PBS (pH 7.4). Wells of a black microtiter plate were loaded with designed different concentrations of peptide solution. Then, 180 µL substrate working solution for trypsin (Z-Gly-Gly-Arg-NHMec•HCl) (Abcam, Cambridge, UK), chymotrypsin (Suc-Ala-Ala-Pro-Phe-NHMec) (Abcam, Cambridge, UK), matriptase (Boc-Gln-Ala-Arg-AMC•HCl) (R&D systems, Minneapolis, MN, USA), mesotrypsin (Mca-Arg-Pro-Val-Glu-Nval-Trp-Arg-Lys(Dnp)-NH_2_) (R&D systems, Minneapolis, MN, USA), and human proteasome 20s (Suc-Leu-Leu-Val-Tyr-AMC) (Abcam, Cambridge, UK) were added and formed a final substrate concentration of 45 μM, 45 μM, 20 μM, 20 μM, and 20 μM, respectively. Blank and control groups were prepared, and trypsin/chymotrypsin/matriptase/mesotrypsin/human proteasome 20s (Sigma-Aldrich, St. Louis, MO, USA; Sigma-Aldrich, St. Louis, MO, USA; R&D systems, Minneapolis, MN, USA; R&D systems, Minneapolis, MN, USA; Biochem, Boston, MA, USA) working solution (10 µL) was added to the wells to initiate hydrolysis reactions. For the human proteasome 20s, florescent was read after 15 min pre-incubation. The fluorescent products’ formation rate was measured continuously at 37 °C in the kinetic model using a multi-detection microplate reader (BMG Labtech, Ortenberg, Germany). The excitation and emission wavelength were set as the manufacturers’ recommendations. Assays were repeated individually three times, and the Ki was calculated by non-linear regression (Morrison equation) using GraphPad Prism.

Minimal inhibitory concentration assay. Antimicrobial activities of the peptides were monitored by determining their minimal inhibitory concentrations (MICs) using three standard microorganisms, which were Gram-positive bacterium *Staphylococcus aureus* (NCTC 10788), Gram-negative bacterium *Escherichia coli* (NCTC 10418), and opportunistic pathogen yeast *Candida albicans* (NCPF 1467). The peptides were dissolved in physiological PBS, two-fold diluted to obtain a series of concentrations, and added into wells of 96-well microtiter plates. The respective concentrations of peptides and controls were incubated with microorganism cultures (5 × 10^5^ colony-forming units/mL) for 18 h at 37 °C in a humidified atmosphere. Then, the growth of microorganisms was detected by measuring optical density at λ = 550 nm using an absorbance reader (BioTek Instruments, Winooski, VT, USA). MIC was defined as the minimum concentration of peptide at which no growth was detectable.

Hemolysis assay. The peptides were dissolved in physiological PBS, two-fold diluted to obtain a series of concentrations, and incubated with a 4% suspension of red blood cells prepared from defibrinated horse blood (TCS Biosciences, Buckingham, UK) for 2 h at 37 °C. The final tested concentrations ranged from 512 μg/mL to 1 μg/mL. Physiological PBS and PBS containing 2% Triton-X100 were used as negative and positive controls, respectively. Lysis of red blood cells was detected by measuring optical density at λ = 550 nm using a plate reader. The hemolytic percentage was calculated using the following formula:

Hemolysis% = (A − A_N_)/(A_P_ − A_N_) × 100%

A was the absorbance of the peptide sample solution. A_N_ and A_P_ were the absorbance of the negative control and positive control, respectively.

Circular dichroism. CD spectra were recorded by the chirascan qCD spectropolarimeter (Applied Photophysics, Surrey, UK). The method referred to a previous publication, with minor modification [32]. Samples were dissolved in DI water at a concentration of 0.5 mg/mL. The spectra were recorded from 190 nm to 250 nm and 250 nm to 320 nm with a 1 nm step resolution, 2 s acquisition duration, 1 nm bandwidth, and 100 mdeg sensitivity. The peptides’ secondary structure composition was predicated and calculated by the BeStSel (Beta Structure Selection) [12].

Molecular simulation and docking. The 3D models of livisin and its analogs were predicted and inferred by using the I-TASSER web server [33], with the crystal structure of HV-BBI (4U2W) [24] as a template. These models’ overall quality was quantified by z-scores using ProSA [34]. We then adopted the bovine trypsin model (4U2W) to simulate the inhibitor–enzyme interaction. The water and ligand molecules in 4U2W were removed, and the missing polar hydrogens were added to the peptide models through Autodock Tools [35]. Followed by energy minimization, the inhibitory peptides were docked to the prepared trypsin model using FlexPepDock, a high-resolution peptide–protein docking tool that was implemented in the Rosetta framework [36]. The confirmation of the simulated molecular docking result with the best affinity score was further optimized by the OPLS-AA force field [37] and was rendered with PyMol (PyMOL Molecular Graphics System, Version 1.8 Schrödinger LLC, New York, NY, USA). The single-point calculations for relative energies were performed using ORCA (RI-B3LYP/def2-SVP) [38]. NCI analyses were conducted using the Multiwfn software to study the relationships between the residues around the active site [14,39,40].

## 5. Conclusions

In conclusion, we identified a novel BBI-like trypsin inhibitory peptide, livisin, from the skin secretion of *O. livida*. The serine-protease-inhibitory activities of livisin and its analogs were assessed and the structure–activity relationship was studied. As aforementioned, peptides in this family have a tremendous function shift, and they possess antimicrobial activity or trypsin-inhibitory activity, or both. The findings provide us with thoughts of identifying novel AMPs or protease inhibitors from animal venoms with drug development potential.

## Figures and Tables

**Table 1:** Ki values of each peptide against the tested proteases.

Peptide	Primary Structure ^1^		Ki (μM)			
		Trypsin	Matriptase	Chymotrypsin	Mesotrypsin	20s Proteasome
livisin	GFLRGC*WTKSFPPKPC*L	2.792	25.15	92.71	22.38	NI ^2^
livisin-NH_2_	GFLRGC*WTKSFPPKPC*L-NH_2_	0.424	2.362	143.8	18.6	NI
livisin-Arg	GFLRGC*WTRSFPPKPC*L	1.016	46.87	74.66	32.08	NI
livisin-Phe	GFLRGC*WTFSFPPKPC*L	NI	NI	3.464	NI	17.72
livisin-loop	C*WTKSFPPKPC*	4.252	NI	NI	54.85	NI

* Represents the cysteine residues that form a disulfide bond in this peptide. ^1^ Shaded residues constitute the inhibitory loop. Residues indicated in red are the modified residues. ^2^ ‘NI’ represents no inhibition. Data were calculated from three independent experiments, each performed in duplicate.

**Table 2:** MIC values of the native livisin and its analogs (μg/mL).

Strains	Livisin	Livisin-NH_2_	Livisin-Arg	Livisin-Phe	Livisin-Loop
*S. aureus* (NCTC 10788)	>512	>512	>512	>512	>512
*E. coli* (NCTC 10418)	>512	>512	>512	>512	>512
*C. albicans* (NCPF 1467)	>512	256	>512	>512	>512

## Data Availability

Not applicable.

## References

[1] Zasloff M. (2002). Antimicrobial peptides of multicellular organisms. Nature.

[2] Wang G., Li X., Wang Z. (2016). APD3: The antimicrobial peptide database as a tool for research and education. Nucleic Acids Res..

[3] Song G., Zhou M., Chen W., Chen T., Walker B., Shaw C. (2008). HV-BBI-A novel amphibian skin Bowman-Birk-like trypsin inhibitor. Biochem. Biophys. Res. Commun..

[4] Conlon J.M., Kim J.B. (2000). A protease inhibitor of the Kunitz family from skin secretions of the tornato frog, Dyscophus guineti (Microhylidae). Biochem. Biophys. Res. Commun..

[5] Wang H., Wang L., Zhou M., Yang M., Ma C., Chen T., Zhang Y., Zeller M., Hornshaw M., Shaw C. (2012). Functional peptidomics of amphibian skin secretion: A novel Kunitz-type chymotrypsin inhibitor from the African hyperoliid frog, Kassina senegalensis. Biochimie.

[6] Wang M., Wang L., Chen T., Walker B., Zhou M., Sui D., Conlon J.M., Shaw C. (2012). Identification and molecular cloning of a novel amphibian Bowman Birk-type trypsin inhibitor from the skin of the Hejiang Odorous Frog; *Odorrana hejiangensis*. Peptides.

[7] Lin Y., Hang H., Chen T., Zhou M., Wang L., Shaw C. (2016). PLR-HL: A Novel Amphibian Bowman-Birk-type Trypsin Inhibitor from the Skin Secretion of the Broad-folded Frog, Hylarana latouchii. Chem. Biol. Drug Des..

[8] Safavi F., Rostami A. (2012). Role of serine proteases in inflammation: Bowman-Birk protease inhibitor (BBI) as a potential therapy for autoimmune diseases. Exp. Mol. Pathol..

[9] Losso J.N. (2008). The biochemical and functional food properties of the Bowman-Birk inhibitor. Crit. Rev. Food Sci. Nutr..

[10] McBride J.D., Watson E.M., Brauer A.B.E., Jaulent A.M., Leatherbarrow R.J. (2002). Peptide mimics of the Bowman-Birk inhibitor reactive site loop. Biopolymers.

[11] Rogers D.M., Jasim S.B., Dyer N.T., Auvray F., Réfrégiers M., Hirst J.D. (2019). Electronic Circular Dichroism Spectroscopy of Proteins. Chem.

[12] Micsonai A., Wien F., Bulyáki É., Kun J., Moussong É., Lee Y.H., Goto Y., Réfrégiers M., Kardos J. (2018). BeStSel: A web server for accurate protein secondary structure prediction and fold recognition from the circular dichroism spectra. Nucleic Acids Res..

[13] De Veer S.J., Li C.Y., Swedberg J.E., Schroeder C.I., Craik D.J. (2018). Engineering potent mesotrypsin inhibitors based on the plant-derived cyclic peptide, sunflower trypsin inhibitor-1. Eur. J. Med. Chem..

[14] Lu T., Chen F. (2012). Multiwfn: A multifunctional wavefunction analyzer. J. Comput. Chem..

[15] Cotabarren J., Broitman D.J., Quiroga E., Obregón W.D. (2020). GdTI, the first thermostable trypsin inhibitor from Geoffroea decorticans seeds. A novel natural drug with potential application in biomedicine. Int. J. Biol. Macromol..

[16] Cotabarren J., Lufrano D., Parisi M.G., Obregón W.D. (2020). Biotechnological, biomedical, and agronomical applications of plant protease inhibitors with high stability: A systematic review. Plant Sci..

[17] Zhou M., Chen T., Walker B., Shaw C. (2006). Lividins: Novel antimicrobial peptide homologs from the skin secretion of the Chinese Large Odorous frog, *Rana (Odorrana) livida*. Identification by “shotgun” cDNA cloning and sequence analysis. Peptides.

[18] Vanhoye D., Bruston F., Nicolas P., Amiche M. (2003). Antimicrobial peptides from hylid and ranin frogs originated from a 150-million-year-old ancestral precursor with a conserved signal peptide but a hypermutable antimicrobial domain. Eur. J. Biochem..

[19] Xu X., Lai R. (2015). The chemistry and biological activities of peptides from amphibian skin secretions. Chem. Rev..

[20] De Veer S.J., Wang C.K., Harris J.M., Craik D.J., Swedberg J.E. (2015). Improving the Selectivity of Engineered Protease Inhibitors: Optimizing the P2 Prime Residue Using a Versatile Cyclic Peptide Library. J. Med. Chem..

[21] Miao Y., Chen G., Xi X., Ma C., Wang L., Burrows J.F., Duan J., Zhou M., Chen T. (2019). Discovery and rational design of a novel bowman-birk related protease inhibitor. Biomolecules.

[22] Lyu P., Ge L., Ma R., Wei R., McCrudden C.M., Chen T., Shaw C., Kwok H.F. (2018). Identification and pharmaceutical evaluation of novel frog skin-derived serine proteinase inhibitor peptide–PE-BBI (*Pelophylax esculentus* Bowman-Birk inhibitor) for the potential treatment of cancer. Sci. Rep..

[23] Zhang L., Chen X., Wu Y., Zhou M., Ma C., Xi X., Chen T., Walker B., Shaw C., Wang L. (2018). A Bowman-Birk type chymotrypsin inhibitor peptide from the amphibian, *Hylarana erythraea*. Sci. Rep..

[24] Grudnik P., Debowski D., Legowska A., Malicki S., Golik P., Karna N., Rolka K., Dubin G. (2015). Atomic resolution crystal structure of HV-BBI protease inhibitor from amphibian skin in complex with bovine trypsin. Proteins Struct. Funct. Bioinform..

[25] Tamura K., Stecher G., Kumar S. (2021). MEGA11: Molecular Evolutionary Genetics Analysis Version 11. Mol. Biol. Evol..

[26] Daly N.L., Chen Y.K., Foley F.M., Bansal P.S., Bharathi R., Clark R.J., Sommerhoff C.P., Craik D.J. (2006). The absolute structural requirement for a proline in the P3′-position of Bowman-Birk protease inhibitors is surmounted in the minimized SFTI-1 scaffold. J. Biol. Chem..

[27] Sreerama N., Manning M.C., Powers M.E., Zhang J.X., Goldenberg D.P., Woody R.W. (1999). Tyrosine, phenylalanine, and disulfide contributions to the circular dichroism of proteins: Circular dichroism spectra of wild-type and mutant bovine pancreatic trypsin inhibitor. Biochemistry.

[28] Gitlin-Domagalska A., Dębowski D., Gucwa K., Starego D., Ptaszyńska N., Sieradzan A., Karczyńska A., Samsonov S.A., Mangold M., Gütschow M. (2020). Truncation of Huia versabilis Bowman-Birk inhibitor increases its selectivity, matriptase-1 inhibitory activity and proteolytic stability. Biochimie.

[29] Brinckerhoff L.H., Kalashnikov V.V., Thompson L.W., Yamshchikov G.V., Pierce R.A., Galavotti H.S., Engelhard V.H., Slingluff C.L. (1999). Terminal modifications inhibit proteolytic degradation of an immunogenic MART-127-35 peptide: Implications for peptide vaccines. Int. J. Cancer.

[30] Dȩbowski D., Łukajtis R., Łȩgowska A., Karna N., Pikuła M., Wysocka M., Maliszewska I., Sieńczyk M., Lesner A., Rolka K. (2012). Inhibitory and antimicrobial activities of OGTI and HV-BBI peptides, fragments and analogs derived from amphibian skin. Peptides.

[31] Tyler M.J., Stone D.J.M., Bowie J.H. (1992). A novel method for the release and collection of dermal, glandular secretions from the skin of frogs. J. Pharmacol. Toxicol. Methods.

[32] Wu D., Gao Y., Wang L., Xi X., Wu Y., Zhou M., Zhang Y., Ma C., Chen T., Shaw C. (2016). A combined molecular cloning and mass spectrometric method to identify, characterize, and design frenatin peptides from the skin secretion of Litoria infrafrenata. Molecules.

[33] Yang J., Yan R., Roy A., Xu D., Poisson J., Zhang Y. (2014). The I-TASSER suite: Protein structure and function prediction. Nat. Methods.

[34] Wiederstein M., Sippl M.J. (2007). ProSA-web: Interactive web service for the recognition of errors in three-dimensional structures of proteins. Nucleic Acids Res..

[35] Österberg F., Morris G.M., Sanner M.F., Olson A.J., Goodsell D.S. (2002). Automated docking to multiple target structures: Incorporation of protein mobility and structural water heterogeneity in autodock. Proteins Struct. Funct. Genet..

[36] London N., Raveh B., Cohen E., Fathi G., Schueler-Furman O. (2011). Rosetta FlexPepDock web server—High resolution modeling of peptide-protein interactions. Nucleic Acids Res..

[37] Jiang F., Zhou C.Y., Wu Y.D. (2014). Residue-specific force field based on the protein coil library. RSFF1: Modification of OPLS-AA/L. J. Phys. Chem. B.

[38] Neese F. (2012). The ORCA program system. Wiley Interdiscip. Rev. Comput. Mol. Sci..

[39] Johnson E.R., Keinan S., Mori-Sánchez P., Contreras-García J., Cohen A.J., Yang W. (2010). Revealing noncovalent interactions. J. Am. Chem. Soc..

[40] Grimme S., Ehrlich S., Goerigk L. (2011). Effect of the damping function in dispersion corrected density functional theory. J. Comput. Chem..

